# The regulation of MMP targeting to invadopodia during cancer metastasis

**DOI:** 10.3389/fcell.2015.00004

**Published:** 2015-02-02

**Authors:** Abitha Jacob, Rytis Prekeris

**Affiliations:** Department of Cell and Developmental Biology, School of Medicine, Anschutz Medical Campus, University of Colorado DenverAurora, CO, USA

**Keywords:** MMP9, MMP2, Rab40B, membrane traffic, invadopodia, cancer, metastasis

## Abstract

The dissemination of cancer cells from the primary tumor to a distant site, known as metastasis, is the main cause of mortality in cancer patients. Metastasis is a very complex cellular process that involves many steps, including the breaching of the basement membrane (BM) to allow the movement of cells through tissues. The BM breach occurs via highly regulated and localized remodeling of the extracellular matrix (ECM), which is mediated by formation of structures, known as invadopodia, and targeted secretion of matrix metalloproteinases (MMPs). Recently, invadopodia have emerged as key cellular structures that regulate the metastasis of many cancers. Furthermore, targeting of various cytoskeletal modulators and MMPs has been shown to play a major role in regulating invadopodia function. Here, we highlight recent findings regarding the regulation of protein targeting during invadopodia formation and function.

## Introduction

Although epithelial cancers are one of the leading causes of death, the mechanisms regulating the development and metastasis of carcinomas are not fully understood. Multiple studies suggest that the progression of tumors is dependent on the intrinsic properties of cancer cells, such as their ability to migrate and invade. Furthermore, many extrinsic factors, such as extracellular matrix (ECM) proteins, are also crucial for regulation of cancer metastasis. The ECM proteins that make up the specialized basement membrane (BM) serve as a barrier for cell invasion. However, the BM which is rich in laminin and collagen IV, also provides the substrate for adhesion of the migrating tumor cells. Furthermore, BM degradation results in the release/activation of various growth factors required for angiogenesis, tumor growth, and metastasis (Kalluri, 2003; Yurchenco, 2011). ECM degrading enzymes known as matrix metalloproteinases (MMPs) are known to play important roles in the degradation of the BM. Since several excellent reviews have already described the importance of MMPs in cancer cell growth and metastasis (Egeblad and Werb, 2002; Deryugina and Quigley, 2006; Fingleton, 2006; Gialeli et al., 2011), this review will focus on the mechanisms governing the targeting of MMPs to invadopodia.

## The role of the family of matrix metalloproteinases (MMPs) in cancer cell metastasis

BM disruption involves a localized degradation of the ECM via the secretion of MMPs (Chamber and Matrisian, 1997). MMPs are a family of zinc endopeptidases that cleave ECM molecules and are subdivided into categories depending on their substrate specificity. The MMP family of enzymes includes not only the classical secreted and membrane bound MMPs, but also ADAMs (a disintegrin and metalloproteinase). ADAM metalloproteinases, also known as sheddases, are involved in cleaving various growth factors, cytokines, receptors, and adhesion molecules and are fundamental to development and homeostasis (Edwards et al., 2008). Like ADAMs, MMPs are also required for normal processes like tissue remodeling in embryonic development, wound healing, involution of mammary glands, angiogenesis, and ossification (Woessner, 1991). However, high levels of MMPs or aberrant MMP expression have often been correlated with pathological conditions like periodontitis, arthritis (Woessner, 1991) and have been implicated in multiple stages of cancer progression including invasion and metastasis (Egeblad and Werb, 2002). In this review, we will focus on the canonical MMPs, more specifically the ones that are targeted to the invadopodia and implicated in BM remodeling during metastasis of epithelial cancers. The association of MMPs with malignancies has been well documented with the majority of the evidence derived from mouse model studies and analysis of human patient samples. Based on substrate recognition, MMPs are categorized into interstitial collagenases, gelatinases, stromelysins, and membrane bound MMPs. Out of the 28 known MMPs, 14 have been implicated in cancer development and progression (Kohrmann et al., 2009). It has been shown that elevated expression of MMP1, 2, 3, 7, 9, 13, and 14 is positively correlated with tumor progression, metastasis, and poor overall prognosis (Lochter et al., 1998; Kerkela and Saarialho-Kere, 2003; Mook et al., 2004; Wagenaar-Miller et al., 2004; Ala-aho and Kahari, 2005; Bjorklund and Koivunen, 2005; Hofmann et al., 2005). Recently, it was shown that MMP9 drives tumor progression and metastasis of triple negative breast cancer (Mehner et al., 2014) and increased expression of MMP9 has been found in the early steps of melanoma (van den Oord et al., 1997). Expression of MMP9 has been associated both positively and negatively with survival rates in breast and colon cancer patients (Zeng et al., 1996; Takeha et al., 1997; Pacheco et al., 1998; Scorilas et al., 2001). Additionally, cancer cells have lesser capability to colonize the lungs of MMP2 or MMP9 deficient mice compared to wild type mice (Itoh et al., 1998, 1999) and cancer cell proliferation is decreased in tumors obtained from MMP9 knock-out mice (Bergers et al., 2000; Coussens et al., 2000). Overexpression of MMP3 and MMP14 (also known as MT1-MMP) has been shown to promote mammary carcinogenesis (Sternlicht et al., 1999; Ha et al., 2001). MMP12 expression in colon cancer has been correlated with increased survival (Yang et al., 2001). These studies indicate that several MMPs play a key role in cancer growth and metastasis. However, the expression levels and functions of individual MMPs are clearly dependent on the stage and type of cancer.

While MMP expression is increased in many cancers, the levels of activated rather than total MMPs appear to be a better indicator of tumor metastatic potential. There are two main mechanisms of post transcriptional regulation of MMP activity: activation of the latent precursor form (zymogen) and inhibition of the active enzyme by tissue inhibitors of MMPs or tissue inhibitors of metalloproteinases (TIMPs) (Polette et al., 2004). Most MMPs are secreted in an inactive pro-enzyme form and are activated extra-cellularly. An interesting property of MMPs is that they are capable of mutual activation. For example, MMP1 and MMP14 can activate MMP2 (Murphy and Crabbe, 1995; Strongin et al., 1995; Sang et al., 1996). The proteolytic activity of MMPs is also regulated by TIMPs, by binding to the zinc ion in their catalytic site (Gomis-Ruth et al., 1997; Fernandez-Catalan et al., 1998). There are four known TIMPs, of which TIMP1 and TIMP2 are the most promiscuous and inhibit the majority of MMPs. *In vivo* studies in mice have shown that overexpression of TIMP1 decreases metastasis to the brain and to the liver (Soloway et al., 1996; Kruger et al., 1997, 1998; Sternlicht and Werb, 2001). Overall, MMP activity is tightly regulated by different mechanisms and is involved in both normal and pathologic processes (Polette et al., 2004).

## The role of invadopodia formation during cancer cell invasion

While the mechanisms mediating the movement of cells through the ECM remain to be fully characterized, it is now widely accepted that the formation of actin rich invasive protrusions is a key step during cancer cell invasion. These structures were identified in tissue culture cells and have been termed as podosomes or invadopodia (Tarone et al., 1985; Chen, 1989). While the functional differences between podosomes and invadopodia remain to be clearly defined, recent nomenclature has tried to distinguish podosomes as present in normal cells and invadopodia as present in cancer cells (Murphy and Courtneidge, 2011; Hoshino et al., 2013). Nevertheless, there are more similarities than differences between podosomes and invadopodia. Both these structures are actin rich and possess the ability to degrade the ECM (Linder and Kopp, 2005) However, they differ in their size, number, lifetime and location, which allows for differentiation between them during visualization (Linder and Kopp, 2005). Both podosomes and invadopodia are usually visualized with phalloidin, which stains filamentous actin and appears as punctate spots located below the nucleus. Podosomes have been observed in cells of monocytic lineage such as macrophages (Lehto et al., 1982; Linder et al., 1999) osteoclasts (Marchisio et al., 1984) and in induced smooth muscle cells (Gimona et al., 2003) as well as endothelial cells (Moreau et al., 2003; Osiak et al., 2005). In contrast, invadopodia are found in cells transformed with oncogenes (David-Pfeuty and Singer, 1980; Tarone et al., 1985) and are thought to protrude further into the matrix and invade more aggressively than podosomes (Weaver, 2008; Linder et al., 2011; Murphy and Courtneidge, 2011). A variety of actin regulators, scaffold proteins, small GTPases, and proteinases have been shown to play important roles in several steps of invadopodia formation. Several studies using animal xenografts and primary tumor cells from patients have also demonstrated the formation of invadopodia *in vivo*. Additionally, invadopodia has been observed in bladder cancer (Sutoh et al., 2010; Yamamoto et al., 2011), colorectal cancer (Schoumacher et al., 2010), breast cancer (Coopman et al., 1998; Yamaguchi et al., 2005a, 2011) squamous cell carcinoma (Takkunen et al., 2010) and glioblastoma (Stylli et al., 2008).

The ability of invadopodia to degrade ECM is attributed to the presence of matrix degrading enzymes such as MMPs. While the cellular machinery mediating the targeted release of MMPs from invadopodia remains to be defined, it is becoming clear that some MMPs are targeted and released from invadopodia to facilitate invasion (Nakahara et al., 1997). MMP14, MMP2, and MMP9 have all been shown to be important in cancer progression and enriched at the invadopodia (Monsky et al., 1993; Nakahara et al., 1997; Bourguignon et al., 1998; Artym et al., 2006; Clark and Weaver, 2008; Poincloux et al., 2009). MMP2 and MMP9 contain fibronectin repeats that help them recognize gelatin (denatured collagen) as a substrate (Polette et al., 2004) and Type IV collagen is the main constituent of the BM, one of the first barriers that cancer cells need to traverse to metastasize. In addition, MMP14 can recognize and cleave a broad spectrum of ECM substrates and also functions as an activator of MMP2 (Lebeau et al., 1999). Thus, the combined activity of MMP2, MMP9, and MMP14 is suggested to be an important step in initiating localized degradation of the BM during epithelial cancer metastasis (Nakahara et al., 1997; Chen and Wang, 1999). Even though this review focuses on the proteolytic aspect of MMP14, it is interesting to note that it can also function through a non-proteolytic mechanism. MMP14 can stimulate ATP production by activating Hypoxia- Inducible Factor-1(HIF-1) (Sakamoto and Seiki, 2010). The non-proteolytic activity of MMP14 also includes binding of its transmembrane domain to integrin β1, which leads to MAPK activation, thereby regulating branching in mammary epithelium (Mori et al., 2013).

Invadopodia formation and function are complex cellular events that involve substantial reorganization in cytoskeleton dynamics and membrane transport. Recent studies have attempted to define different stages of invadopodia formation and function by using various criteria, including the recruitment of actin, targeted release of MMPs and the localized degradation of the ECM. Based on these, invadopodia formation has been divided into three stages, namely, initiation, assembly, and maturation. The following sections describe the stages of invadopodia formation in detail.

### Initiation

In the initiation phase of invadopodia formation, invadopodial precursors, or “buds” form at the cell periphery which are usually marked by actin puncta (Yamaguchi et al., 2005b). The process of invadopodia formation is initiated by growth factors such as epidermal growth factor (EGF), platelet derived growth factor (PDGF) and transforming growth factor- β (TGFβ). Growth factor receptor signaling activates Phosphatidylinositide 3-Kinase (P13K) leading to Src activation, which in turn phosphorylates multiple proteins including Tks5 (Tyrosine kinase substrate). Since the PX domain (phospholipid binding domain) of Tks5 has been shown to bind to PI3P and PI(3,4)P_2_ (Abram et al., 2003), it was suggested that Tks5 localizes to PI(3,4)P_2_ enriched regions of the plasma membrane, thus targeting Tks5 to initiate the invadopodia “bud” (Courtneidge et al., 2005). Src phosphorylates synaptojanin 2 to activate its phosphatase activity, which dephosphorylates PI(3,4,5)P_3_at the plasma membrane to form PI(3,4)P_2_, thus forming the site for invadopodia formation (Chuang et al., 2012). Src mediated activation of the Abl-family kinase Arg also leads to the phosphorylation of cortactin, resulting in the recruitment of Nck1 to the invadopodia (Oser et al., 2009; Mader et al., 2011). Nck1 then recruits the Neural Wiskott-Aldrich syndrome protein (N-Wasp) complex to the invadopodia leading to Cdc42-dependent activation of N-Wasp. N-Wasp in turn induces actin polymerization through the Arp2/3 complex, resulting in formation of invadopodial precursors (Yamaguchi et al., 2005a). The co-localization of cortactin with Tks5 in invadopodial “buds,” led to the hypothesis that Tks5 acts a scaffolding protein that recruits the other cellular components required for initiation of invadopodia formation. However, some recent evidence suggests that Tks5 might instead be involved in invadopodia maturation (Sharma et al., 2013).

### Assembly

Invadopodia are highly dynamic and motile structures that have been divided into two types based on their motility and lifetimes, motile short-lived and stationary long-lived invadopodia (Yamaguchi et al., 2005a). The short-lived invadopodia are thought to be precursors of fully functional mature invadopodia and could also be equivalent to podosomes (Yamaguchi et al., 2005a). *In vitro*, newly formed or early invadopodia can move laterally within the plasma membrane that faces the ECM. These motile invadopodia are then anchored and stabilized by actin polymerization and extension of the invadopodia (Yamaguchi et al., 2005a). A plethora of proteins are recruited to the invadopodial “bud” converting it from motile to stationary invadopodia. The precise order of how proteins are recruited is still unknown. Since Tks5 has five tandem SH3 domains, it is thought that Tks5 can scaffold several proteins like Nck1, Nck2, Grb2 (Growth factor receptor bound protein 2) and N-Wasp. It has also been proposed that Tks5, along with cortactin, recruits various actin regulators leading to nucleation of branched actin filaments and the formation of a stable actin core in the invadopodia (Clark and Weaver, 2008; Oser et al., 2009). Consistent with this hypothesis, it has been shown that phosphorylation of cortactin leads to dissociation of the cortactin/cofilin complex, which is an essential step in invadopodia formation and elongation (Oser et al., 2009). The dissociation of the cortactin/cofilin complex and the polymerization of actin is also a pH-dependent process (Pope et al., 2004; Frantz et al., 2008).

### Maturation

Actin polymerization is crucial for formation and function of invadopodia. Inhibition of cofilin results in the formation of short-lived unstable invadopodia, which suggests that actin polymerization caused by cofilin is required for elongation and maturation of invadopodia (Yamaguchi et al., 2005a). Apart from the branched actin network, invadopodia also contain linear actin bundles (Li et al., 2010; Schoumacher et al., 2010). mDia2, a formin that induces the formation of linear actin networks, has been found to promote elongation and stability of invadopodia (Lizarraga et al., 2009). Fascin, another actin bundling protein has also been shown to promote elongation, stability and matrix degradation in invadopodia (Li et al., 2010; Schoumacher et al., 2010).

Src kinase is a major regulator of invadopodia formation and function. Interestingly, several other protein kinases including Abl kinases like Arg (Abl related gene) kinase have recently emerged as important players in invadopodia formation and maturation (Beaty et al., 2013). It was shown that β1 integrin interacts with Arg leading to stimulation of Arg mediated cortactin phosphorylation, a key switch in promoting invadopodial maturation (Beaty et al., 2013). Additionally, β1 integrin has been shown to localize to invadopodia and promote degradation of collagen type IV, the main constituent of the BM (Sameni et al., 2008), presumably by recruiting and docking proteases at the invadopodia. Separase, a gelatinolytic enzyme that has been shown to be enriched at the invadopodia, binds to α3β1 integrin resulting in the formation of functional invadopodia (Mueller et al., 1999). The ultrastructure of mature invadopodia has shown the presence of microtubules and many vesicles/endosomes indicative of active trafficking and a possible route for delivery of specific proteins like MMPs (Schoumacher et al., 2010). The activity of proteases docked at the invadopodia has been shown to be pH-dependent (Greco et al., 2014). Furthermore, it was demonstrated that the acidification of the peri-invadopodial space by the Na^+^/H^+^ exchanger (NHE1) promotes ECM proteolysis (Busco et al., 2010).

## Mechanisms mediating MMP targeting to invadopodia

The final maturation stage of the invadopodia involves targeted delivery and exocytosis of MMP2, MMP9, and MMP14. The appearance of these MMPs is generally considered to be a mark of functional mature invadopodia. As the result, much effort has been invested in understanding the regulation of MMP targeting to invadopodia, leading to recent studies defining the machinery governing MMP transport during cancer cell invasion.

### Targeting of MMP14

**Figure 1:**
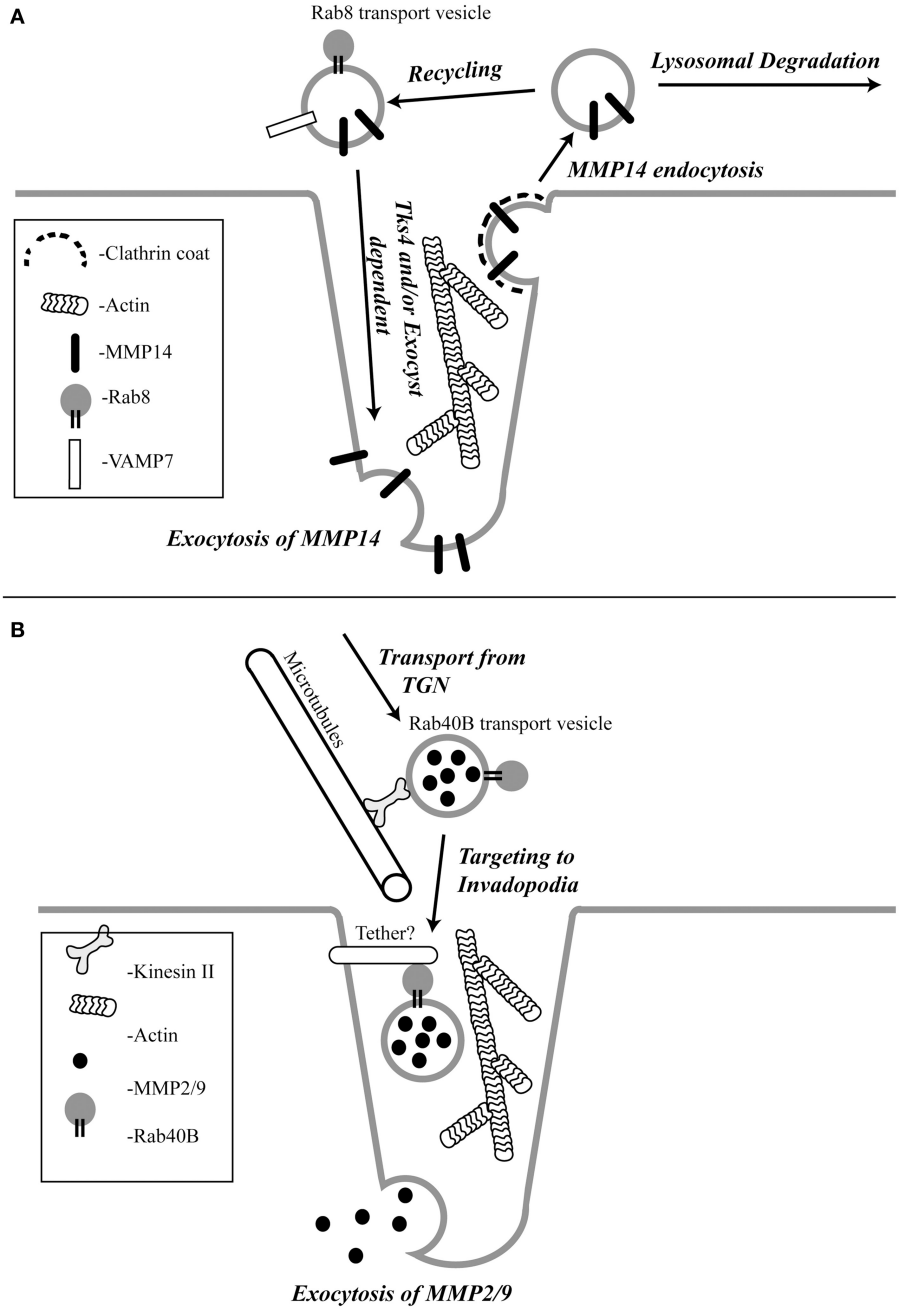
**The schematic representation of the pathways regulating MMP14 (A) and MMP2/9 (B) targeting to the invadopodia**.

MMP14 is a membrane embedded MMP whose extracellular proteolytic activity is regulated by a balance between exocytosis and internalization via clathrin and/or caveolar mediated endocytosis (Remacle et al., 2003; Figure 1A). Once internalized, MMP14 is then either targeted to lysosomes for degradation (Jiang et al., 2001; Remacle et al., 2003) or shunted to endocytic recycling pathways, thus controlling the levels of active enzyme at the cell surface (Remacle et al., 2003; Figure 1A). However, invasive cancer cells have mechanisms to counteract the removal of the active enzyme from the plasma membrane. Consistently, enrichment of active MMP14 at the invadopodia associated plasma membrane of tumor cells has been demonstrated *in vitro* (Nakahara et al., 1997; Artym et al., 2006; Clark and Weaver, 2008; Steffen et al., 2008). Endocytic recycling (Poincloux et al., 2009) exocytosis (Monteiro et al., 2013), Rab 8 (Bravo-Cordero et al., 2007) and Tks4 (Buschman et al., 2009) have all been shown to be involved in the localization of MMP14 to the invadopodia. However, the exact mechanism governing MMP14 targeting remains to be fully understood. Rab8 GTPase has been shown to be involved in mobilization of MMP14 from intracellular storage compartments allowing polarized recruitment of MMP14 to the invasive front of cells (Bravo-Cordero et al., 2007). Some of the exocytic components shown to regulate delivery of MMP14 to the invadopodia are cortactin, the Exocyst complex (consists of 8 subunits) and VAMP7. Cortactin has been shown to regulate the cell surface expression of MMP14 (Clark et al., 2007). Sec8, a subunit of the Exocyst complex, was shown to localize at the invadopodia and is required for MMP14 targeting to the invadopodia (Monteiro et al., 2013; Figure 1A). Active RhoA and Cdc42 trigger the interaction between the Exocyst subunits Sec3 and Sec8 and the polarity protein IQGAP1. This interaction has been shown to be required for the accumulation of MMP14 at the invadopodia (Sakurai-Yageta et al., 2008). The Exocyst complex has also been shown to interact with Arp2/3 activator Wiskott-Aldrich syndrome protein and Scar homolog (WASH) to ensure focal delivery of MMP14 to the invadopodia (Monteiro et al., 2013). Since exocytosis depends on SNAREs (soluble N-ethylmaleimide sensitive factor attachment protein receptors) which drive the fusion of transport vesicles with the plasma membrane, several recent studies have investigated the role of SNAREs in mediating MMP14 transport. Consequently, it was shown that VAMP7 (vesicle associated membrane protein 7) is required for trafficking of MMP14 to invadopodia (Steffen et al., 2008; Figure 1A).

Transport vesicle targeting and fusion with its destination membranes often relies on specific tethering factors that impart specificity to membrane transport. The tethering factors regulating MMP14 targeting remain to be identified. However, Tks4, a scaffolding factor related to Tks5, has been shown to be required for the formation and function of invadopodia (Buschman et al., 2009). In the absence of Tks4, recruitment of MMP14 to podosomes is inhibited, implicating the role of Tks4 in targeting of MMP14 to the invadopodia (Buschman et al., 2009; Figure 1A). Additionally, cortactin was reported to have a novel role in invadopodial maturation and invasion by regulating secretion of MMP14 at the invadopodia (Clark and Weaver, 2008). However, it remains to be tested whether Tks4 and cortactin function as MMP14-vesicle tethers, or whether they affect MMP14 targeting indirectly by regulating the actin cytoskeleton within during invadopodia formation and maturation. Recently, it was also shown that Orai1 calcium channels can also regulate MMP14 targeting, since Orail1-mediated Ca^2+^ oscillations regulate MMP14 recycling to the plasma membrane (Sun et al., 2014).

### Targeting of MMP2 and MMP9

MMP2 and MMP9 are gelatinases that possess fibronectin type II repeats that allow them to degrade collagen and gelatin. Gelatinolytic degradation can cause the release of signaling molecules from the ECM that aid cell migration and angiogenesis. A lot of effort has been focused on understanding the transport and targeting of gelatinases because they are overexpressed in a variety of tumors and are associated with tumor aggressiveness and poor patient prognosis (Pacheco et al., 1998; Egeblad and Werb, 2002; Hiratsuka et al., 2002; van 't Veer et al., 2002). Although MMP2 and MMP9 have been shown to be enriched at the invadopodia, there is not much known about how these proteases are transported and targeted to the invadopodia. It has been reported that MMP2 and MMP9 are stored and transported in small vesicles that move along microtubules powered by kinesin in human melanoma cells (Schnaeker et al., 2004; Figure 1B). Similar to MMP14, the secretion of MMP2 and MMP9 were also shown to depend on cortactin (Clark and Weaver, 2008). Interestingly, in contrast to MMP14, endocytic transport and the Exocyst complex do not appear to play a role in targeted transport of MMP2 and MMP9 (Jacob et al., 2013). Instead, MMP2 and MMP9 are transported via specialized secretory vesicles directly from the trans-Golgi Network (TGN) to the invadopodia (Jacob et al., 2013). While much of the machinery mediating the formation and transport of these secretory vesicles remains to be defined, it was recently shown that Rab40b GTPase plays an important role in targeting MMP2/9 to the invadopodia (Jacob et al., 2013; Figure 1B).

## Invadopodia formation and function *in vivo*

Although there is an increasing amount of evidence demonstrating the existence of invadopodia *in vitro*, the formation and function of invadopodia *in vivo* is less well understood due to challenges associated with visualizing and distinguishing these structures in animal models. Cancer invasion usually occurs deep in tissues and these events are highly dynamic and unpredictable making it difficult to visualize invadopodia during primary tumor metastasis. Though the majority of invadopodial studies have been conducted in 2D tissue culture systems, some groups have studied invadopodia formation in 3D matrices as they better simulate the physiological *in vivo* environment. Such studies of invadopodia in complex 3D matrices have shown that the matrix degrading activity is localized to the base rather than the tip of the invadopodia (Wolf et al., 2007; Tolde et al., 2010). These 3D studies have also helped establish criteria for the identification of invadopodia *in vivo* and provide a good model to study formation of invadopodia.

Despite the challenges mentioned above, there is some compelling evidence drawn from elegant *in vivo* experiments that confirm that invadopodia are not just *in vitro* artifacts. Recently, the chorioallantoic membrane of the chicken embryo was used to visualize the intravascular formation of invadopodia and the extravasation of tumor cells into the stroma (Leong et al., 2014). The same group also showed that knocking down invadopodial components like cortactin, Tks4 and Tks5 decreases extravasation of cells into the lung stroma in tail vein injected mice. Intravital live animal imaging has also allowed the visualization of MtLn3-GFP (a highly invasive rat mammary carcinoma line) invading into blood vessels using protrusions identified as invadopodia-like structures using invadopodia markers such as cortactin and N-WASP (Lohmer et al., 2014). Using live-cell imaging, it was shown that during the uterine-vulval development in *Caenorhabditis elegans*, the anchor cell breaches the uterine and vulval basement membranes by making an invadopodium (Hagedorn et al., 2013). The Src-Tks5 pathway was shown to be required for the migration of neural crest cells using actin-rich protrusions in zebrafish (Murphy et al., 2011). Finally, it has also been shown that the intestinal epithelium of the zebrafish mutant *meltdown* forms invadopodia-like protrusions that invade into the stromal tissue in response to cues from the surrounding smooth muscle layer (Seiler et al., 2012).

## Conclusions and future directions

Significant advances have been made in understanding the formation and function of invadopodia. However, there are still a lot more unknowns regarding this subcellular structure. While all of the above mentioned studies have helped to confirm the physiological role of invadopodia as a structure used by invasive cells to penetrate the basement membrane, more evidence is required to elucidate the functional role of invadopodia *in vivo* and understand how widespread the use of invadopodia by cells is. Many questions regarding the importance of invadopodia in cancer invasion and metastasis still exist. Future studies in the field of invadopodia will need to focus on detection of invadopodia in human cancer samples as well as to identify the role of invadopodia in the different steps of the metastatic cascade. The other areas that require focus are the identification of components specific to invadopodia that can be targeted and the development of compounds that can specifically inhibit invadopodia formation and function. These issues will need to be addressed before invadopodia can become a candidate for development of new cancer therapies.

### Conflict of interest statement

The authors declare that the research was conducted in the absence of any commercial or financial relationships that could be construed as a potential conflict of interest.
